# Management and outcomes of sigmoid volvulus: a retrospective cohort study of 46 patients at a tertiary care center

**DOI:** 10.1097/RC9.0000000000000467

**Published:** 2026-04-06

**Authors:** Nida Khan, Rafique U. Harvitkar, Lorcan Moore, Premjithlal Bhaskaran, Heena Patel, Christi Swaminathan

**Affiliations:** a Department of General Surgery, Royal Sussex County Hospital, University Hospitals Sussex NHS Foundation Trust, Brighton, UK; b Department of General Surgery, St Richard’s Hospital, University Hospitals Sussex NHS Foundation Trust, Chichester, UK; c Department of Cardiovascular Surgery, Imperial College, London, UK

**Keywords:** decompression, flexible sigmoidoscopy, Hartmann’s procedure, ischemia, volvulus

## Abstract

**Introduction:**

Sigmoid volvulus (SV) is a potentially life-threatening cause of large bowel obstruction that requires prompt diagnosis and intervention to reduce the morbidity and mortality rates. This study evaluated the clinical characteristics and outcomes of patients presenting with SV at a tertiary care center.

**Presentation of case:**

We retrospectively reviewed the records of all patients who presented with SV between October 2020 and September 2025. Demographic data, comorbidities, imaging, endoscopic decompression, surgical intervention, length of hospital stay, postoperative complications, and 30- and 100-day mortality were also recorded.

**Discussion:**

Forty-six patients were identified (29 men and 17 women; mean age, 77.67 years). All patients underwent initial imaging with abdominal radiography or CT, followed by flexible sigmoidoscopy for decompression. Eighteen patients underwent surgical management, whereas 28 were managed conservatively. The operative group had a longer mean hospital stay (29.18 ± 26.02 days SD) than the non-operative group (12.66 ± 16.52 days SD). Postoperative complications occurred in 28% of the surgically treated patients. No 30-day deaths occurred in the operative group, although two 100-day deaths were recorded in the same group. In comparison, the nonoperative group had nine 30-day deaths and five 100-day deaths, largely among patients with high ASA scores, comorbidities, frailty, and neurological impairment.

**Conclusion:**

SV predominantly affected elderly patients with comorbidities in this cohort. Although surgery was associated with longer hospitalization, it demonstrated lower short-term mortality than conservative management. Early clinical stratification and timely definitive intervention may improve outcomes but must be balanced against the risks associated with operating on frail geriatric patients.

## Introduction

Volvulus is a twisting of the intestine and mesentery that supplies it^[^1^]^. The sigmoid colon is the most common site of volvulus owing to its tendency to elongate, and in some individuals, the presence of a redundant mesentery with a narrow posterior attachment to the retroperitoneum. It may also occur in patients with a hypermobile cecum and rarely in the transverse colon. Volvulus can obstruct venous outflow, arterial inflow, or both, eventually leading to intestinal ischemia and even gangrene^[^2^]^. In the UK, sigmoid volvulus (SV) accounts for 2%–3% of all cases of intestinal obstruction and 5%–10% of large bowel obstructions^[^3^]^.

HIGHLIGHTS

- Sigmoid volvulus is predominantly seen in elderly patients, particularly those with frailty, neuro-psychiatric disorders and diabetes.
- Endoscopic decompression is the initial treatment of choice but recurrence during the same admission is very common.
- Surgical intervention offers definite treatment.
- Surgery demonstrates lower short-term mortality compared to conservative management.
- Non-operative management carried significantly higher 30- and 100-day mortality rates.
- Surgery is not always feasible as a significant number of these patients are too frail, co-morbid and with high ASA scores.
- Early risk stratification and individualized decision-making are essential for optimal outcomes.

Patients with SV typically present with pronounced abdominal distension, constipation, and abdominal tenderness. Abdominal radiography is diagnostic and classically demonstrates the “coffee-bean” sign, an inverted U-shaped loop^[^4^]^. Characteristic features on computed tomography of the abdomen and pelvis (CTAP) include the whorl pattern of a dilated sigmoid loop around the mesocolon and its vessels and a bird-beak appearance of the afferent and efferent limbs^[^5^]^.

All patients required detorsion with flexible sigmoidoscopy following initial resuscitation, except in cases where bowel ischemia or perforation is suspected^[^6^]^. Approximately 25% of patients require urgent surgical intervention due to failed endoscopic decompression, colonic ischemia, or perforation^[^7^]^. In an emergency setting, Hartmann’s procedure is often appropriate, whereas resection with primary anastomosis may be considered for elective presentations of recurrent SV^[^8^]^.

Given that a significant proportion of patients presenting with SV are elderly, frail, and have comorbidities, management can be challenging. Many patients are unfit for or may decline surgical intervention. This study aimed to evaluate the outcomes of different treatment modalities for SV and identify the risk factors associated with morbidity and mortality in a tertiary center cohort.

## Material and methods

This retrospective observational cohort study included 46 consecutive patients who presented with SV between October 2020 and September 2025 at a tertiary care NHS trust. Patients who underwent emergency endoscopic decompression, emergency surgical intervention, or elective surgery for recurrent volvulus were included, with follow-up extending up to 1 year after the index presentation. SV was diagnosed through imaging and confirmed by endoscopy. Patients who were misdiagnosed with colonic pseudo-obstruction were excluded from the analysis.

All patients initially underwent resuscitation, followed by flexible sigmoidoscopy for endoscopic decompression as first-line management. Surgical intervention was considered for recurrent volvulus, failed endoscopic decompression, or suspected ischemia or perforation. Operative interventions included Hartmann’s procedure, sigmoid resection with primary anastomosis (with or without diverting ileostomy), loop colostomy, or sigmoidopexy, with the choice of intervention informed by bowel viability, frailty, comorbidities, and clinical stability. Data were extracted from hospital electronic records and included patient demographics, comorbidities, Rockwood Clinical Frailty Score, imaging findings, time to endoscopic intervention, recurrence, need for surgery, procedure type, and postoperative complications.

The outcomes assessed included 30-day and 1-year mortality in those who reached 1-year follow up, length of hospital stay, recurrence of volvulus, failure of endoscopic decompression, and postoperative morbidity. Comparisons were made between patients who were managed conservatively and those who underwent surgery. Statistical analysis was performed using standard statistical software. Welch’s *t*-test to compare the length of hospital stay between the two groups and Fisher’s exact test to compare the mortality rates, with a significance threshold set at *P* < 0.05.

This work has been reported in lines with PROCESS criteria^[^9^]^.

This study was conducted as a registered clinic audit at Royal Sussex County Hospital, University Hospitals Sussex NHS Foundation Trust. As the project involved retrospective analysis of anonymized patient data collected during routine clinical care, formal Research Ethics Committee approval was not required according to NHS governance policy. This study was conducted in accordance with the principles of the Declaration of Helsinki.

## Results

A total of 46 patients were diagnosed with SV during the study period, with 17 females (36%) and 29 males (63%), and a mean age of 77.67 years; notably, 78% were aged ≥70 years. CTAP was the primary imaging modality in 30 patients (65%), abdominal radiography in 15 (33%), and one case was identified incidentally during CT colonography. All patients underwent endoscopic decompression, with 36 (78%) successfully decompressed and six (13%) experiencing failed or partial decompression. Four patients (8.7%) demonstrated ischemic changes, and recurrence during the same admission occurred in 15 patients (33%). Thirty-two procedures (70%) were performed within 24 h, whereas 12 (26%) occurred after 24 h, although procedural data were incomplete for two patients.

The comorbidities are listed in Table 1.

**Table 1:** Co-morbidities in patients with sigmoid volvulus.

Comorbidity	Number of patients (*n*)
Pulmonary disorders	3
Cardiac disorders	15
Hypertension	13
Neurological disorders	22
Chronic kidney disease	3
Psychiatric disorders	7
Diabetes mellitus (DM)	5
Hypothyroidism	5
Others	20

Eighteen patients (39%) underwent surgical management, of which 15 (83.3%) were emergency procedures and 3 (16.7%) were elective. The indications included recurrent volvulus after at least two endoscopic decompressions (15 patients), failed endoscopic decompression (1 patient), colonic ischemia (1 patient), and colonic perforation (1 patient). Postoperative complications occurred in five patients (28%), with two Clavien–Dindo grade II and three grade IV events requiring critical care. The mean length of stay was significantly longer in the operative group (29.18 ± 26.03 days SD) than in the non-operative group (12.66 ± 16.52 days SD), excluding two private operative cases and four inpatient deaths. Welch’s *t*-test confirmed statistical significance (*P* = 0.0239). Mortality outcomes differed markedly between groups: no 30-day deaths were reported in the surgical cohort, and two 100-day deaths occurred due to metastatic prostate cancer and urosepsis. In contrast, the nonoperative group had nine 30-day deaths (32%), all ASA III/IV, and an additional five deaths by 100 days. Fisher’s exact test demonstrated significantly higher 30-day (*P* = 0.0074) and 100-day (*P* = 0.009) mortality rates in the conservatively managed group.

The surgical interventions are listed in Table 2.

**Table 2:** Surgical interventions in patients with sigmoid volvulus.

Surgical procedure	Number of patients (*n*)
Open Hartmann’s procedure	8
Laparoscopic Hartmann’s procedure	3
Loop colostomy	1
Laparoscopic sigmoidopexy	1
Total colectomy	1
Sigmoid colectomy with anastomosis	4

Subgroup analysis showed that, of 19 patients with a single episode of SV, four (21%) underwent surgery for recurrence in same admission, perforation, and mucosal necrosis. Of the ten patients with two presentations, one required sigmoid colectomy. Among the 17 patients with three or more presentations, 14 (82%) underwent surgery. The remaining four patients were managed conservatively owing to significant frailty (Rockwood 6–7, ASA III), stability without recurrence, or patient choice. One frail patient died from a chest infection within 30 days, and another experienced recurrent volvulus after laparoscopic sigmoidopexy and was deemed unfit for further surgery.

## Discussion

SV is commonly observed in elderly patients^[^10^]^. Atamanalp *et al* reported a mean age of 71.1 years, which is similar to the mean age of 77.67 years in our cohort^[^11^]^. Previous studies have demonstrated a strong male predominance, with Emna *et al* reporting a male-to-female ratio of 5.4:1^[^12^]^. Our cohort showed a male-to-female ratio of 3:2, indicating a higher incidence in males. This difference has been attributed to sex-specific anatomical variations, including dolichosigmoid anatomy in males and brachiosigmoid configuration in females, the latter characterized by a wider mesentery that may reduce torsion^[^13^]^. Additionally, a wider pelvis and laxer abdominal wall in females may facilitate spontaneous detorsion^[^14^]^. SV is also associated with chronic constipation, mental health disorders, Alzheimer’s disease, Parkinson’s disease, and Hirschsprung’s disease^[^15^]^. Diabetes mellitus is reported to complicate 2.6%–41.1% of SV cases; in our study, five patients (10%) were diabetic^[^16^]^.

Patients with peritoneal signs suggest underlying ischemia, gangrene, or perforation. CTAP is particularly valuable when the diagnosis is uncertain or when ischemia or perforation is suspected, with a diagnostic accuracy of up to 89%^[^5^]^. In our cohort, 33% underwent abdominal radiography, and 65% underwent CT imaging. The standard first-line management for uncomplicated SV involves flexible endoscopic decompression without bowel preparation, followed by the placement of a flatus or rectal tube. Recurrence after endoscopic decompression is well documented and may be as high as 90%^[^17^]^. In our cohort, recurrence during the same admission occurred in 33% of the patients.

Surgery remains a critical component in the management of SV, particularly in cases of ischemia or perforation^[^18^]^. The 30-day mortality in the nonoperative group was 32%, comparable to the 37.2% reported by Moro-Valdezate *et al*^[^19^]^. Abdelrahim *et al* found that mortality in the conservatively managed group was confined to patients with ASA III–IV status and Rosa *et al* reported a high mortality rate of 42% in non-operatively managed SV, consistent with our findings^[^3^]^. Our postoperative complication rate of 28% aligns with the 27% reported by Lee *et al*, supporting the view that while surgery carries considerable morbidity, it offers substantially improved survival compared with conservative management in appropriately selected patients^[^20^]^.

## Conclusion

This retrospective cohort study highlights the complexities of SV. Endoscopic decompression remains first-line, but definitive surgical management is associated with lower short-term mortality in appropriately selected patients. Operative decisions depend on the patient’s frailty and comorbidities. An individualized multidisciplinary approach remains key to optimizing outcomes in these patients.

## Data Availability

Data will be made available upon reasonable request.

## SCARE checklist

This case series has been reported in line with the SCARE 2020 criteria.
